# Meta-analysis reveals complex marine biological responses to the interactive effects of ocean acidification and warming

**DOI:** 10.1002/ece3.516

**Published:** 2013-03-07

**Authors:** Ben P Harvey, Dylan Gwynn-Jones, Pippa J Moore

**Affiliations:** 1 Institute of Biological, Environmental, and Rural Sciences, Aberystwyth UniversityAberystwyth, UK, SY23 3DA; 2 Centre for Marine Ecosystems Research, Edith Cowan UniversityPerth, Australia, 6027

**Keywords:** Climate change, meta-analysis, multiple stressors, ocean acidification, ocean warming, synergistic interactions

## Abstract

Ocean acidification and warming are considered two of the greatest threats to marine biodiversity, yet the combined effect of these stressors on marine organisms remains largely unclear. Using a meta-analytical approach, we assessed the biological responses of marine organisms to the effects of ocean acidification and warming in isolation and combination. As expected biological responses varied across taxonomic groups, life-history stages, and trophic levels, but importantly, combining stressors generally exhibited a stronger biological (either positive or negative) effect. Using a subset of orthogonal studies, we show that four of five of the biological responses measured (calcification, photosynthesis, reproduction, and survival, but not growth) interacted synergistically when warming and acidification were combined. The observed synergisms between interacting stressors suggest that care must be made in making inferences from single-stressor studies. Our findings clearly have implications for the development of adaptive management strategies particularly given that the frequency of stressors interacting in marine systems will be likely to intensify in the future. There is now an urgent need to move toward more robust, holistic, and ecologically realistic climate change experiments that incorporate interactions. Without them accurate predictions about the likely deleterious impacts to marine biodiversity and ecosystem functioning over the next century will not be possible.

## Introduction

The concentration of atmospheric carbon dioxide (CO_2_) has increased from 280 ppm in preindustrial times to a present day level of 391 ppm (Le Quéré et al. 2012). Over the last 100 years, this has led to changes in global sea surface temperatures (+0.74°C) and ocean carbonate chemistry (Orr et al. 2005), which have included ocean acidification by 0.1 pH units (Caldeira and Wickett 2003; Kleypas et al. 2006). By the year 2100, sea surface temperatures are expected to rise by a further 1–4°C while increased CO_2_ (aq) will result in the decreased availability of carbonate ions and a further reduction in pH by 0.3–0.5 units (Caldeira and Wickett 2005; IPCC 2007; Gooding et al. 2009). These changes in temperature and ocean carbonate chemistry are considered two of the greatest threats to marine biodiversity (Kleypas et al. 1999; Doney et al. 2009), leading to changes in the physiological performance of individual organisms, which will in turn alter biotic interactions, community structure, and ecosystem functioning.

A range of marine biological responses have already been observed in response to ocean warming including hypoxia (Pörtner and Knust 2007), coral bleaching (Hoegh-Guldberg et al. 2007), species range shifts (Parmesan and Yohe 2003; Root et al. 2003), changes to phenology (Walther et al. 2002), and reduced organism body size (Daufresne et al. 2009). Experimental manipulations simulating predicted future ocean temperatures have suggested that warming will also lead to increased metabolic costs for plants and animals (O'Connor et al. 2009), increased consumption rates (Sanford 1999), and changed food-web structures (Petchey et al. 1999). Observed responses of marine organisms to recent ocean acidification are limited (but see Iglesias-Rodriguez et al. 2008b; Moy et al. 2009), but are expected to become increasingly apparent in the next 50–100 years (Doney et al. 2009; Feely et al. 2009). Experimental evidence, however, suggests that responses are likely to be varied (Hendriks et al. 2010; Kroeker et al. 2010) and will include hypercapnic suppression of metabolism (Christensen et al. 2011), acid–base balance disturbances (Miles et al. 2007), plus both positive and negative effects on skeleton formation (related to a decrease in carbonate saturation; Doney et al. 2009; Ries et al. 2009).

The vulnerability of marine species and ecosystems to temperature, in particular, is well established (for reviews; Hoegh-Guldberg and Bruno 2010; Richardson et al. 2012; Wernberg et al. 2012). Conversely, the resilience of marine organisms to ocean acidification still remains a reasonably challenged concept (Dupont et al. 2010a; Hendriks and Duarte 2010). Recent meta-analyses assessing the biological effects of ocean acidification (Dupont et al. 2010a; Hendriks et al. 2010; Kroeker et al. 2010) concurred that it is unlikely to act in a uniform manner as variation exists in marine organism responses and resilience. Hence, if any meaningful comparisons are to be made on the response of marine organisms, they need to be hypothesis driven based on a priori assigned groupings, such as taxonomic groups or life stages (Dupont et al. 2010a).

Studies of the biological effects of elevated temperature and acidification on marine organisms in isolation have provided some insight into the potential tolerance of species to these changing conditions (Gattuso and Hansson 2009). However, given that these stressors are unlikely to operate independently (Halpern et al. 2007), there is now a need to gain a more ecologically realistic understanding of how the combined effects of temperature and acidification will affect marine biota (Sala et al. 2000; Fabry et al. 2008). This is vital in order to inform future adaptive management strategies. Other recent meta-analyses, across ecological systems, have also shown that multiple stressors can lead to nonadditive interactions with responses dependent on the type of stressor as well as the level of ecological organization investigated (e.g., population vs. community, autotroph vs. heterotroph) (Crain et al. 2008; Darling and Côté 2008; Tylianakis et al. 2008). Moreover, the mechanism through which the stressor acts upon the organism will affect the response. Multiple stressors acting through a similar pathway may have an additive effect (Crain et al. 2008). In contrast, any stress-induced tolerances could lead to antagonisms (Blanck 2002), while those stressors that act on different, but dependent mechanisms may act synergistically (Kneitel and Chase 2004). These reviews did, however, contain few, if any, studies that investigated both warming and acidification. Therefore, the concurrent effect of temperature and ocean acidification via elevated CO_2_ remains unclear, but is likely to lead to complex biological outcomes.

Organisms vary widely in their individual responses to ocean warming and acidification as a result of differences in their physiological and ecological characteristics (Dupont et al. 2008; Fabry 2008). For example, many marine organisms possessing a calcium carbonate (CaCO_3_) structure would be considered more susceptible to ocean acidification as this process will impair their capacity to produce calcified skeletons (Doney et al. 2009). Conversely, some species, including some calcified species, will have the capacity to buffer against the deleterious effects of acidification by utilizing acid–base compensation (Claiborne and Evans 1992; Larsen et al. 1997), active mobility and metabolism (Widdicombe and Spicer 2008; Whiteley 2011) or energy reallocation (Wood et al. 2008; McDonald et al. 2009). Warmer temperature, up to a limit, stimulates metabolism in ectotherms, resulting in faster growth and development (Byrne 2011). Moreover, it has been speculated that warming could even ameliorate the negative impacts of acidification (McNeil et al. 2004; Kleypas and Yates 2009).

Species responses to ocean warming and acidification are also likely to vary among life-history stages (Byrne 2011). Early life-history stages are considered most susceptible to changes in both temperature and ocean acidification (Byrne 2011). These stressors may, however, have positive and/or negative effects for the successful recruitment of juveniles to the adult population. Trophic level is also likely to determine how species respond due to differences in environmental sensitivity (Petchey et al. 2004; Raffaelli 2004). Previous study has suggested that the effects of multiple stressors are likely to act antagonistically in autotrophs, but synergistically in heterotrophs (Crain et al. 2008). Furthermore, as higher trophic levels contain less “biological insurance” (sensu Yachi and Loreau 1999), that is, less taxonomic, physiological, and genetic diversity, they are predicted to be more susceptible to multiple environmental perturbations (Christensen et al. 2006) which could act upon them synergistically (Crain et al. 2008).

Using a meta-analytical approach of the peer-reviewed literature, we assessed the impacts and interactions of ocean acidification and warming on marine biological responses. Given that variability in the strength and direction of responses was expected, we classified data according to taxonomic groups, calcifiers and noncalcifiers, life-history stage and level of trophic organization (autotroph and heterotroph) in terms of changes in rates of calcification, growth, photosynthesis, reproduction, and survival. Specifically, we aimed to address three questions: (i) How do marine organisms respond to warming and acidification in isolation? (ii) How do marine organisms respond to the combined effects of warming and acidification? (iii) How do warming and acidification impacts interact?

## Material and Methods

### Data selection and suitability criteria

Searches for peer-reviewed articles in which studies explicitly investigated climate change using either elevated temperature, ocean acidification or elevated temperature and acidification were carried out using ISI Web of Science [v. 5.8] © and Google Scholar using the following keywords: ocean warming, global warming, ocean acidification, hypercapnia, climate change, and combinations therein. In addition, we used the European Project on Ocean Acidification (EPOCA) blog (http://oceanacidification.wordpress.com/), citation searches; analysis of reference lists in comprehensive reviews (Hendriks et al. 2010; Kroeker et al. 2010; Wernberg et al. 2012), and then cross-referenced with the bibliographies of identified articles.

We limited our analysis to studies published between 1st January 1990 and 1st January 2012, as the majority of experimental climate change studies that manipulated climate change conditions in line with IPCC AR1 predictions and subsequent updates (IPCC 1990, 2007) were published post 1990. Only controlled manipulative experiments were used for analysis. In addition, the control treatments of the environmental stressors (e.g., pH, CO_2_, or temperature) needed to represent current ambient levels and were based on the authors' opinion of “ambient”. The experimental organisms had to be subjected to elevated temperature alone, acidification alone, or both warming and acidification. When studies included environmental variables in addition to temperature and ocean acidification (such as light availability or nutrients), these responses were only considered at “ambient” levels as determined by the authors'. To explore predicted future conditions for 2100, the manipulation treatments needed to conform to the IPCC IS92a “business-as-usual” emission scenario for the year 2100 (IPCC 2007). We omitted studies that manipulated carbonate chemistry using acid addition, because it does not reproduce the changes in HCO_3_^-^ concentration that occur as a result of increased CO_2_ (aq) (Iglesias-Rodriguez et al. 2008a,b). Finally, only studies that reported a measureable biological response were included.

As response variables, we used calcification (or dissolution) rates, growth, photosynthesis, reproduction, and survival (mortality was converted to survival by using 1 – mortality). There were insufficient data on other response variables (e.g., feeding rates, metabolism) to allow quantitative analysis. A number of articles included more than one species, response, location, or treatment level and all were included in the analysis if they met the suitability criteria. This ensured that a broad range of responses could be fully explored, despite lessening the independence of the data from that particular study (Gurevitch et al. 1992). To maintain independence of data, we included only one response, chosen at random, from studies reporting several responses that could be classified in the same category (e.g., growth expressed as changes in length and biomass). Derived metrics from studies that included time-series data were based on the final time point of exposure. To investigate inherent biological variability, records were categorized according to taxonomy, life-history stage, level of trophic organization (autotroph, heterotroph), and whether the organism possessed a CaCO_3_ skeletal structure.

To enable a calculation of effect size, studies that met our initial criteria could only be used if they reported a mean response value, some form of variance (standard deviation, standard error, or confidence interval), and a sample size. In some instances, data were extracted from graphical images in publications and in these situations data were extracted using the program GraphClick (v. 3.0) (Neuchatel, Switzerland).

### Data analysis

Biological responses to ocean warming and acidification were measured for each experiment to establish the proportional change between the control and treatment means using response ratios. In their original metric response ratios were weighted toward positive responses, so the response ratios were log transformed to maintain symmetry in the analysis and ease the biological interpretation (Hedges et al. 1999). We chose a log response ratio (lnRR), over other methods, to estimate the effect size because of the high capacity to detect true effects and their robustness to small sample sizes (Lajeunesse and Forbes 2003).

Analyses were carried out using the R (version 2.15.1; R Development Core Team 2012) package “Metafor” (Viechtbauer 2010). We selected a weighted random-effects model to estimate a summary effect size. Random-effects analysis assumes that the true effect size differs between experiments and the estimated summary effect is the mean of the effects observed across the studies. This meant that even if studies had a low weighting, the individual effect sizes from all of the studies could be incorporated into the summary effect (Borenstein et al. 2009). This ensured that the biological variation inherent in the responses was properly accounted for. Both the within-study variance (inverse of the effect size variance) and the between-study variance (σ^2^_pooled_) were used to weight the studies. Therefore, studies with higher replication and/or lower variance were considered more precise and weighted accordingly (Hedges and Olkin 1985). Between-study variance was estimated using the DerSimonian Laird method (DerSimonian and Laird 1986).

Statistical significance was attributed to each summary effect size by calculating a bias-corrected 95% confidence interval (CI) (see Hedges and Olkin 1985) and comparing it with zero. If the summary effect size did not overlap zero then it was considered to be significantly different. A total heterogeneity statistic (*Q*) was used to ascertain that the variation observed was a combination of both true variation (between studies) and random error (within studies) (Borenstein et al. 2009). This was tested as the observed weighted sum of squares against a chi square distribution with *n*−1 degrees of freedom, using the null hypothesis that observations shared a common effect size.

Combinations of the treatment effect (CO_2_/pH, temperature, temperature and CO_2_/pH) and response variables (calcification, growth, photosynthesis, reproduction, and survival) were used as the comparison groups in all analyses. Separate exploratory analyses were also used to test the differences between a priori defined groups using a mixed-effects model (see Viechtbauer 2010). It was appreciated that this form of multiple exploratory analyses on the same dataset could be prone to Type I error. Hence, we used these analyses to identify the underlying patterns of the biological responses. The categorical moderators used were the different taxonomic groups (corals, crustaceans, crustose coralline algae, echinoderms, fishes, noncalcifying algae, molluscs, phytoplankton, and seagrasses), calcifying and noncalcifying organisms, developmental stages (embryos, larvae, juveniles, and adults), and trophic organization (autotroph and heterotroph). This process applied a summary effect size and 95% CI to each of the different categories for comparison. To formally test for differences between these categories, a test for heterogeneity (*Q*_*M*_) was used; this identified total heterogeneity explained by that particular categorical moderator (Gurevitch et al. 1992). A significant *Q*_*M*_ indicated that there was a difference between the categories.

The taxonomic group of phytoplankton was initially divided into coccolithophores, cyanobacteria, diatoms, dinoflagellates, and foraminifera, however, results were pooled after detecting no differences using a test for heterogeneity (*Q*_*M*_). When assigning a “trophic organization” to each observation, we defined autotrophs as any organism capable of producing organic carbon-based compounds from inorganic sources, through either photosynthesis or chemosynthesis. For example, corals were designated as autotrophic as photosynthesis is generally regarded as their principal mode of carbon acquisition (Hoogenboom et al. 2006; Mass et al. 2007). Over all of the meta-analytical results, the summary effect sizes were not reported if there were fewer than five studies available for analysis, and categorical moderators were not reported if there were fewer than three studies. This was a pragmatic decision to ensure that a broad range of responses could be assessed, as some categories only had a few studies that met our criteria. Therefore, the categorical analyses did not always include all the observations from the full model.

### Interactions between multiple stressors

Interaction strength between ocean warming and acidification was ascertained according to the methods for factorial meta-analysis (Gurevitch et al. 2000; Crain et al. 2008). To be included, studies needed to be controlled factorial experiments reporting four outcomes of acidification [*Y*_acid_], warming [*Y*_temp_], acidification and warming [*Y*_both_], and a control treatment [*Y*_ct_] (Underwood 1997). Therefore, not all the observations from the full model could be analyzed. Multiple observations from the same study were included if separate factorial results were provided. The interaction strength (lnRR_int_) and individual effects (lnRR_acid_ and lnRR_temp_) of each study were then calculated as


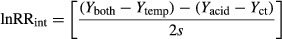



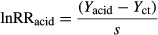



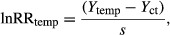


where *Y* is the biological response for the indicated group (in subscript), and *s* is the pooled standard deviation. The sampling variance of lnRR_int_ is


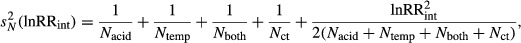


where *N* is the sample size for the indicated group (in subscript).

Although less conservative than an additive model (Folt et al. 1999), we used a multiplicative model to calculate the interactions as the underlying model of the metric lnRR is multiplicative (Hawkes and Sullivan 2001; Morris et al. 2007), and this model is also thought to be more biologically realistic (Sih et al. 1998). Synergisms occur when the cumulative response of a stressor (*Y*_both_ − *Y*_temp_) is greater than the stressor in isolation (*Y*_acid_ − *Y*_ct_), and antagonistic when the cumulative impact is smaller than expected (Folt et al. 1999). The interaction strength (lnRR_int_) was then synthesized into a summary effect for each type of biological responses using a random-effects model with the same methodology as outlined previously. Therefore, the interaction type was classified as multiplicative (i.e., the null hypothesis) if the interaction effect size and 95% CI overlapped zero. If the direction of both individual stressors was positive, the interaction was considered synergistic when the interaction effect size was greater than zero, and antagonistic if less than zero. Similarly, when the direction of the individual stressors was either both negative or had opposite signs, then the interpretation was reversed (i.e., synergies occurred when the interaction effect size was less than zero).

### Sensitivity analyses and publication bias

Sensitivity analysis was used to investigate the influence of any experimental study that demonstrated an unusually large effect size. This was achieved in a stepwise manner by ranking each experiment by the magnitude of effect size, removing the largest one, and rerunning the analysis. Likewise, if any study contributed five or more observations to a category, the study was omitted and the analyses were rerun. If studies were considered to be driving the results, then they were omitted from the analysis of that response variable.

The number of studies with an effect size of zero that would be required to change the results of the meta analysis from significant to nonsignificant (“file drawer problem”) was determined using Rosenthal's fail-safe number (Rosenthal 1979). It was decided that if five or less studies (of zero effect size) were required to change the effect size, then that categorical analysis was not considered robust.

## Results

### Overall biological responses

Out of 196 peer-reviewed articles that investigated the biological responses of marine organisms to ocean warming and/or acidification 107 met our criteria, giving 623 unique observations (Table S1). Observations that did not meet the selection criteria are listed in Table S2, and the results from all the heterogeneity tests for overall within effects (*Q*) and between categories (*Q*_*M*_) are reported in Table S3.

**Figure 1:**
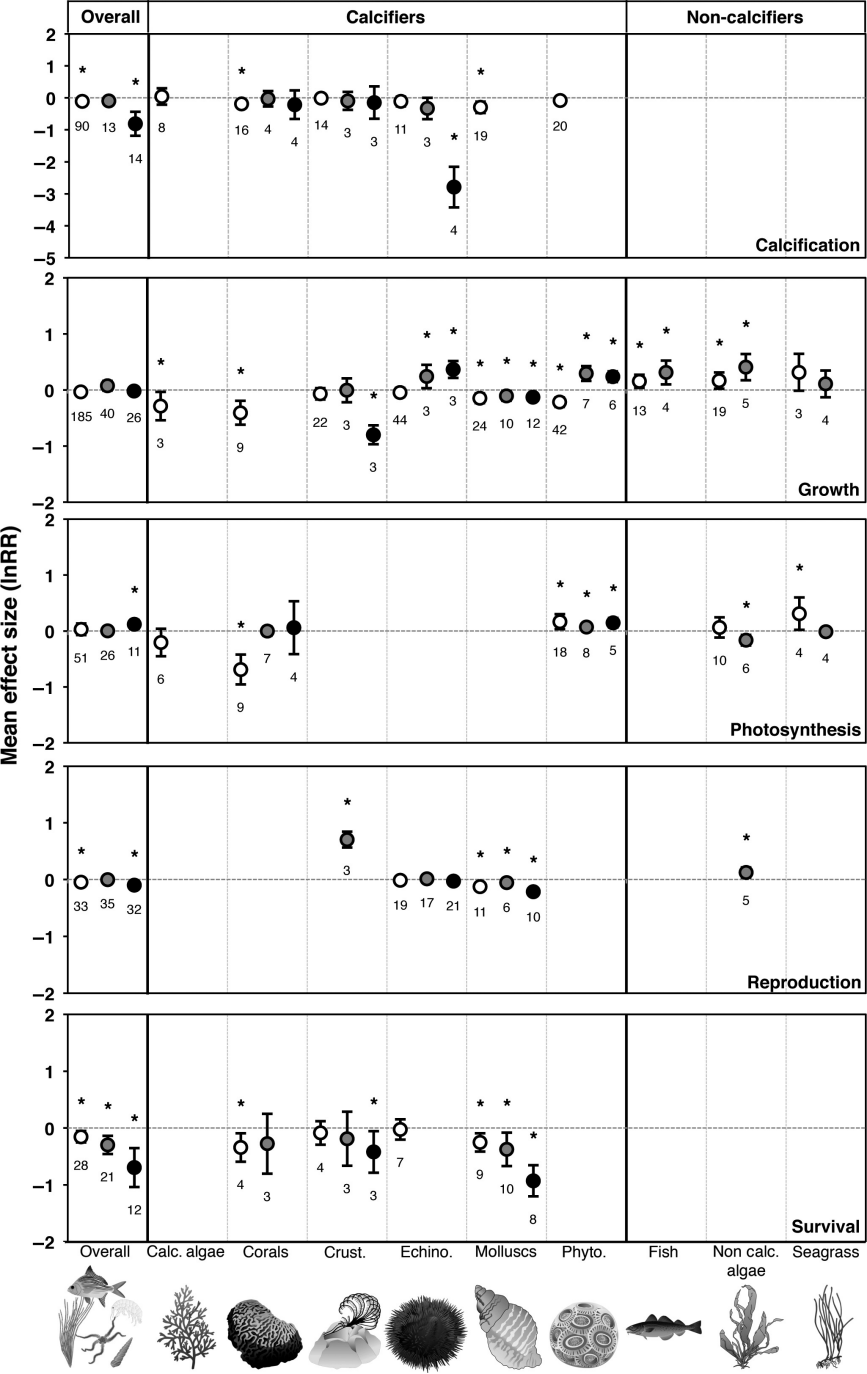
The mean effect of ocean acidification (clear circles), ocean warming (gray circles), and combined ocean acidification and warming (black circles) on calcification, growth, photosynthesis, reproduction, and survival for different taxonomic groups. The mean log response ratio and ±95% confidence intervals are shown for overall (combined results), calcifiers (calcifying algae, corals, crustaceans, echinoderms, molluscs, and phytoplankton) and noncalcifiers (fishes, noncalcified algae, seagrass). The number of observations in each analysis is shown in parentheses. The zero line indicates no effect, and significance (*) of mean effects is determined when the ±95% confidence interval does not overlap zero.

Meta-analysis of the whole dataset revealed that calcification and reproduction were negatively affected by ocean acidification and neutrally affected by ocean warming. In contrast, the independent effects of ocean acidification and warming had no effect on growth or photosynthesis, while both ocean acidification and warming had significant negative effects on survival (Fig. 1).

Combined warming and acidification had a significant negative effect on calcification, survival, and reproduction with the magnitude of response greater than that observed for the stressors in isolation (Fig. 1). Concurrent acidification and warming had no effect on growth overall, but had a significant positive effect on photosynthesis in autotrophs (Fig. 1).

Significant within-group heterogeneity was observed in all the responses (Table S3), therefore in order to quantify patterns in the literature regarding the effects of warming and acidification, we made comparisons between the a priori groupings (taxonomic groups, calcifiers and noncalcifiers, life-history stage, and level of trophic organization) for each biological response.

#### Calcification

Although the independent effects of ocean acidification and warming on calcification were varied, we did not detect differences between taxonomic groups (Fig. 1; Table S3), life-history stages (Fig. 3; Table S3), or trophic organization (Fig. 4; Table S3). Conversely, the combined effects of ocean warming and acidification on calcification varied significantly by taxonomic group (Fig. 1; *P <* 0.001; Table S3), with echinoderms more negatively affected than either corals or crustaceans (both *P* < 0.001); life-history stage (Fig. 3; *P* = 0.0397; Table S3) with juveniles more negatively affected compared with adults (*P* = 0.040); and trophic organization (Fig. 4; *P* = 0.010; Table S3) with heterotrophs being negatively affected by warming and acidification, while autotrophs were neutrally affected (Fig. 4).

#### Growth

**Figure 2:**
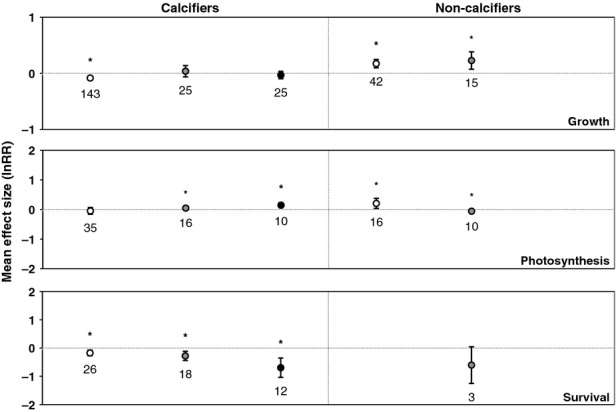
The mean effect of ocean acidification (clear circles), ocean warming (gray circles), and combined ocean acidification and warming (black circles) on growth, photosynthesis, and survival for calcifying and noncalcifying organisms. The mean log response ratio and ±95% confidence intervals are shown for calcifiers and noncalcifiers. The number of observations in each analysis is shown in parentheses. The zero line indicates no effect, and significance (*) of mean effects is determined when the ±95% confidence interval does not overlap zero.

**Figure 3:**
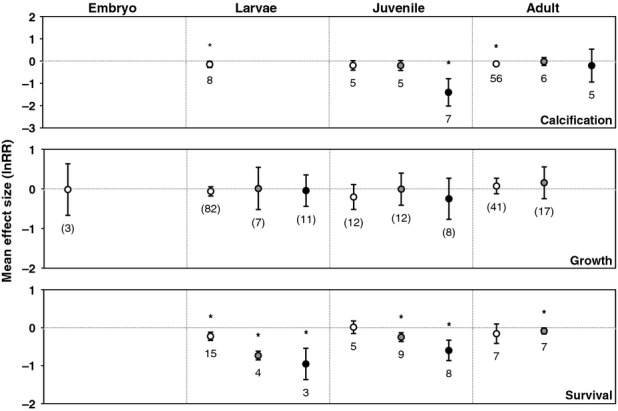
The mean effect of ocean acidification (clear circles), ocean warming (gray circles), and combined ocean acidification and warming (black circles) on calcification, growth, and survival in different life-history stages. The mean log response ratio and ±95% confidence intervals are shown for embryos, larvae, juveniles, and adults. The number of observations in each analysis is shown in parentheses. The zero line indicates no effect, and significance (*) of mean effects is determined when the ±95% confidence interval does not overlap zero.

**Figure 4:**
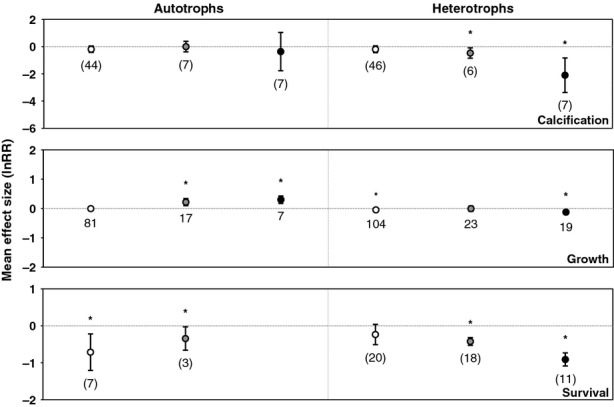
The mean effect of ocean acidification (clear circles), ocean warming (gray circles), and combined ocean acidification and warming (black circles) on calcification, growth, photosynthesis, reproduction, and survival for different levels of trophic organization. The mean log response ratio and ±95% confidence intervals are shown for autotrophs and heterotrophs. The number of observations in each analysis is shown in parentheses. The zero line indicates no effect, and significance (*) of mean effects is determined when the ±95% confidence interval does not overlap zero.

Despite overall growth responses being neutrally affected by ocean acidification and warming, we detected differences in the combined effects between taxonomic groups (Fig. 1; *P* < 0.001; Table S3). Crustaceans and molluscs were both significantly negatively affected, while echinoderms and phytoplankton were positively affected (Fig. 1). The combined effects of warming and acidification varied with trophic organization (Fig. 4; *P* < 0.001; Table S3), with autotrophs being positively affected and heterotrophs negatively affected (Fig. 4). Similarly, ocean warming also varied between taxonomic groups (Fig. 1; *P* < 0.001; Table S3), with echinoderms, phytoplankton, fishes, and macroalgae positively affected, and molluscs negatively affected (Fig. 1); and trophic organization (Fig. 4; *P* = 0.004; Table S3) with a positive effect on autotrophs and a neutral effect on heterotrophs. Ocean warming also positively affected noncalcifiers (Fig. 2). The independent effects of ocean acidification varied by taxonomic group (Fig. 1; *P* < 0.001; Table S3), with crustose coralline algae, corals, molluscs, and phytoplankton negatively affected, crustaceans and echinoderms unaffected, and fishes, macroalgae, and seagrass positively affected (fishes and macroalgae *P* < 0.05, seagrass *P* = 0.06); and calcifiers/noncalcifiers (Fig. 2; *P* < 0.001; Table S3) with the calcifying organisms exhibiting generally negative effects and noncalcifiers generally positive effects (Figs. 1, 2). No differences in growth responses were detected between life stages with any of the stressors (Fig. 3).

#### Photosynthesis

We did not detect differences in the effects of combined warming and acidification on photosynthesis between either taxonomic groups (corals and phytoplankton; Fig. 1), calcifiers/noncalcifiers (Fig. 2), or life stages (Fig. 3), despite finding significant differences between these a priori groupings in both acidification and warming independently (Table S3). We did, however, find that in combination significant positive effects were observed in phytoplankton (Fig. 1) and calcifying organisms (Fig. 2). Similarly, ocean warming positively affected photosynthesis in phytoplankton and calcifying organisms, but had a negative effect on both macroalgae (Fig. 1) and noncalcifying organisms (Fig. 2). The independent effects of ocean acidification on photosynthesis also varied by taxonomic group (Fig. 1; *P* < 0.001; Table S3), with corals being negatively affected, and both phytoplankton and seagrass positively affected. Ocean acidification also positively affected photosynthesis in noncalcifying organisms (Fig. 2).

#### Reproduction

The effects of warming and acidification, both combined and independently, on reproduction were varied between the taxonomic groups (Fig. 1; all *P* < 0.001; Table S3). In response to ocean warming alone positive responses by crustaceans and macroalgae and autotrophs were observed while heterotrophs responded negatively (*P* = 0.0071, Table S3). In all three combinations of stressors, echinoderms were unaffected while molluscs were negatively affected.

#### Survival

The combined effects of warming and acidification resulted in significant negative responses across the categories, but only varied by taxonomic group (Fig. 1; *P* = 0.030; Table S3). Specifically, combined acidification and warming had a larger negative effect on molluscs than crustaceans (Fig. 1; *P* = 0.029). Independently, the effects of ocean warming on survival also varied by life stage (Fig. 3; *P* < 0.001; Table S3) with a larger negative effect on juveniles compared with adults (*P* = 0.023) and on larvae compared with both juveniles and adults (both *P* < 0.001). Additionally, ocean warming also had a negative effect on molluscs (Fig. 1), calcifiers (Fig. 2), and both autotrophs and heterotrophs (Fig. 4). Survival responses to ocean acidification in isolation did not vary between taxonomic group, calcifiers/noncalcifiers, life stage, or trophic organization. However, ocean acidification did negatively affect corals and molluscs (Fig. 1), calcifiers (Fig. 2), larvae (Fig. 3), and autotrophs (Fig. 4).

### Interactions between multiple stressors

Interaction strength between ocean warming and acidification was determined for the subset of fully factorial studies, and therefore did not include all the observations from the full model. Significant synergistic interactions were observed for calcification (*z =* 3.69, *P* < 0.001), photosynthesis (*z =* 2.23, *P* = 0.026), reproduction (*z =* 2.97, *P* = 0.003), and survival (*z =* 3.04, *P* = 0.002), but not for growth (*z =* 1.56, *P* = 0.117).

### Sensitivity analyses and publication bias

To test the robustness of our analyses against large effect sizes, we removed each comparison stepwise and reran each analysis, omitting experiments if they changed the significance of either heterogeneity or the mean effect size of the response variables. This resulted in 12 experiments being omitted from subsequent analyses across several treatment-response variable scenarios (see Table S2 for more detail). We used Rosenthal's fail-safe number to assess the importance of potential publication bias and found that our response variables were robust, with the lowest value being 358 additional studies required to change the effect size (based on an original experiment quantity of 11). No individual study contributing more than five experiments changed the significance of either the heterogeneity or mean effect size of the response variables.

## Discussion

This study used a meta-analytical approach to assess the impacts and interactions of ocean acidification and warming on marine biological responses. Responses were classified according to taxonomic groups, calcifiers and noncalcifiers, level of trophic organization (autotroph and heterotroph), and life-history stage in terms of changes in rates of calcification, growth, photosynthesis, reproduction, and survival. We assessed marine organisms responses to warming and acidification in isolation and in combination and assessed the biological implications of their interaction.

### Effects of ocean acidification

Ocean acidification generally had an adverse effect on a large range of marine biota with more specific differences between life-history characteristics. Calcifying organisms were generally negatively affected by ocean acidification, with significant biological variability in responses, such as molluscs being negatively affected, which may be due to their poor ion regulation and inability to buffer their internal compartments (Fabry et al. 2008; Widdicombe and Spicer 2008; Melzner et al. 2009; Dupont et al. 2010a). Conversely, crustaceans were generally unaffected by acidification perhaps due to their active mobility, higher metabolism, and capacity to control intracellular pH (Gaillard and Malan 1983; Widdicombe and Spicer 2008; Whiteley 2011).

Overall, less is known about how noncalcifying organisms are likely to respond to acidification (Connell and Russell 2010), particularly marine fishes (Ishmatsu et al. 2008; Munday et al. 2009), however, our results show that noncalcifiers were generally unaffected by acidification and for growth were positively affected when analyzed together as a group. Fishes are thought to have more efficient acid–base regulation compared to invertebrates (Widdicombe and Spicer 2008), which, when coupled with an increased food intake or reduced energy expenditure (Munday et al. 2009), could explain the positive growth response observed. Similarly, noncalcifying marine autotrophs demonstrated an increased growth to ocean acidification. This is possibly via their capacity to derive dissolved inorganic carbon from the increased CO_2_ (aq) (Beer and Koch 1996).

Autotrophs may be capable of increasing their inorganic carbon assimilation (Rost and Riebesell 2004) and buffering the negative effects on calcification (Ries et al. 2009). However, negative effects on growth were observed in calcifying autotrophs. Our results suggest that the negative effects on calcifying autotrophs may outweigh positive effects associated with the increased availability of CO_2_ (aq) as a substrate for photosynthesis. We also show some evidence that calcification and survival in early life-history stages were more negatively affected by ocean acidification, highlighting not only the susceptibility of early life stages, but also the subtle nature of life-history responses when compared to species-specific effects of heterogeneity (Kurihara 2008; Kroeker et al. 2010). Importantly, our results agree with the findings from previous meta-analyses (Dupont et al. 2010a; Kroeker et al. 2010) while introducing a further 48 key studies (168 additional data points).

### Effects of ocean warming

Moderate elevations in temperatures will increase metabolic rates (Hochachka and Somero 2002), which influences key biological processes that regulate life-history characteristics (O'Connor et al. 2007). While marine organisms are capable of acclimation to a range of temperatures, once their thermotolerance limits are exceeded, organism fitness is reduced and the risk of mortality increases (Hofmann and Todgham 2010; Tomanek 2010). We found that both noncalcifying organisms and autotrophs demonstrated increased growth under warming conditions, likely due to this increase in metabolic rate (Hochachka and Somero 2002). Warming, however, had no effect on the growth of heterotrophs and even negatively affected their calcification. Possibly because the metabolism complexes of autotrophs (photosynthesis-limited) are less sensitive to ocean warming than the respiration-limited metabolism of heterotrophs (Lopez-Urrutia et al. 2006). Moreover, our results also support the hypothesis that the threshold for deleterious warming may vary between developmental stages (Byrne et al. 2009, 2010) with survival significantly lower in larvae than in juveniles, and significantly lower in juveniles than in adults.

### Simultaneous acidification and warming

Meta-analysis of the full dataset revealed that the combined stressors caused significant negative effects on calcification, reproduction, and survival, and a significant positive effect on photosynthesis, but no effect on growth. Importantly, we also found that four of the five responses (calcification, photosynthesis, reproduction, and survival) showed a synergistic interaction between acidification and warming. Although, such synergistic interactions between stressors (i.e., where the outcome was greater than the sum of the individual stressors; Folt et al. 1999) are relatively common (e.g., Sala et al. 2000; Harley et al. 2006), they are concerning because they are also unpredictable. Hence, such synergies limit our capacity to predict potential future impacts from single-stressors studies.

Ecological synergies are important to marine systems (Paine et al. 1998; Harley et al. 2006; Sutherland et al. 2006) because they can further exacerbate adverse effects and reduce ecosystem resilience (Folke et al. 2004). They can also introduce indirect effects via biotic interactions (Darling and Côté 2008; Tylianakis et al. 2008). For example, climate-driven changes in plankton communities can regulate top predators through bottom-up control (Beaugrand et al. 2003). Moreover, as marine systems are subject to multiple interacting stressors (Halpern et al. 2007), it is possible that the addition of further stressors would introduce additional adverse consequences (e.g., Przeslawski et al. 2005). Therefore, our results highlight the need to move away from single-stressor studies and toward more ecologically realistic research incorporating multiple stressors, in order to more fully understand how near-future anthropogenic change will affect marine biodiversity.

There was, as expected, variation in our analyses among the biological responses to combined warming and acidification between different taxonomic groups, calcifiers and noncalcifiers, trophic levels, and life-history stages. The combination of warming and acidification generally exhibited a stronger effect (either positive or negative) than when exposed to the stressors in isolation. For instance, echinoderms are highly vulnerable to ocean acidification (Dupont et al. 2010b), likely due to their skeletons being formed from highly soluble magnesium calcite (Politi et al. 2004). However, the addition of moderate increases in temperature (+4°C), as predicted for the end of the century, resulted in further adverse effects (e.g., Byrne et al. 2011), such as the highly negative calcification responses shown here. In some instances, the combined effects of warming and acidification were reduced compared with the individual effects. Corals had both calcification and photosynthesis negatively affected by ocean acidification in isolation, while they were unaffected by the combined effects, suggesting that the addition of warming may ameliorate the adverse effects of acidification (McNeil et al. 2004; Kleypas and Yates 2009). These differences in the resilience of marine organisms will have important implications for ecosystem level responses.

Interestingly, the combined effect of warming and acidification positively affected growth in echinoderms, which could be explained by energy allocation, where the cost of homeostatic regulation can be influenced by changes in somatic and reproductive growth performance (Melzner et al. 2009). Hence, if more energy is utilized to maintain growth, then calcification responses could be more adversely affected (e.g., Arnold et al. 2009; McDonald et al. 2009). Other studies have shown an alternative strategy for energy allocation where growth was negatively affected, while calcification was maintained (e.g., Wood et al. 2008; or crustaceans, this study), highlighting the species-specific nature of biological responses.

Heterotroph responses to ocean acidification and warming individually differed, with acidification reducing growth, but not affecting calcification or survival; while warming alternatively did not affect growth, reducing both calcification and survival responses. However, the energetic demands of the combined effects of acidification and warming on heterotrophs resulted in calcification, growth, and survival all being reduced. Conversely, the combined effects of warming and acidification positively affected growth in autotrophs, likely due to the effect of temperature on metabolic rate (Hochachka and Somero 2002), while CO_2_, acted as a substrate for photosynthesis and possibly indirectly promoted growth (e.g., phytoplankton; Loehle 1995). Moreover, as multiple stressors affecting autotrophs are likely to act antagonistically (Crain et al. 2008), and the dissolved inorganic carbon sources utilized by marine autotrophs is set to increase (Raven 2005), photosynthesizing organisms will likely be more resilient to conditions predicted for the end of the century; as long as they do not exceed their thermotolerance (Hofmann and Todgham 2010; Tomanek 2010) and are not limited by other factors, such as inorganic nutrient availability (Langdon and Atkinson 2005; Cohen and Holcomb 2009; Ries et al. 2009).

Our results also show that the combined effects of warming and acidification had significant negative effects on the survival of early life-history stages. Although there was insufficient data to compare across all life-history stages, both larvae and juveniles were highly susceptible to changes in temperature and ocean acidification, supported by previous research (Gosselin and Qian 1997; Hunt and Scheibling 1997; Byrne 2011). Indirectly, ocean warming can reduce the mortality of larvae by shortening the planktonic duration (Lamare and Barker 1999), when they are most vulnerable to predation (O'Connor et al. 2007). Our results, however, identified that the combined effects of ocean acidification and warming increased mortality, indicating that multiple stressors will have important implications for population persistence, potentially acting as a bottleneck for some species (Dupont et al. 2010b; Byrne 2011).

## Conclusions

Quantitative syntheses of the published literature can provide powerful inferences, however, like all analyses they are subject to caveats. We identified and incorporated the available literature that met our selection criteria, however, this also outlines the current gaps in knowledge and highlights opportunities for further study. Moreover, species-specific sources of heterogeneity are always likely to make some results from the literature greatly context dependent (e.g., Fabry 2008; Kurihara 2008; Dupont et al. 2010a; Hendriks et al. 2010; Kroeker et al. 2010). Hence, our findings highlight the complexity of marine organism responses to ocean warming, acidification, and their interaction. The magnitude, direction, and interaction of the effects varies between response types, likely a result of the pathways driving the biological responses. Responses also differ between taxonomic groups, trophic levels, and life-history stages. Most importantly, we observed synergistic interactions between ocean acidification and warming in four of the five biological responses measured (calcification, photosynthesis, reproduction, and survival), highlighting the difficulties in making inferences from single-stressor studies. However, single-factor studies in junction with those that manipulate multiple stressors can play a vital role in understanding the pathways through which particular stressors operate and will enable a more accurate assessment of the likely outcomes of interactions between warming and acidification. Importantly, we must also consider further abiotic and biotic stressors in the marine environment that are likely to also interact with warming and acidification (Halpern et al. 2007) as well as scaling up studies from individuals and populations to communities and ecosystems (Harley et al. 2006). Such large-scale multifactorial experiments would not only increase our knowledge of the functioning and resilience of marine ecosystems, but provide explicit evidence to policymakers on the effectiveness of conservation and management strategies in response to future environmental change.

## Supporting Information

**Table S1.** Experiments included in meta-analysis.

**Table S2.** Selection criteria for exclusion from meta-analysis.

**Table S3.** Heterogeneity tests – within groups (*Q*) and between groups (*Q*_*M*_).
